# Direct and Long-Term Metabolic Consequences of Lowly vs. Highly-Digestible Starch in the Early Post-Weaning Diet of Mice

**DOI:** 10.3390/nu10111788

**Published:** 2018-11-17

**Authors:** José M. S. Fernández-Calleja, Lianne M. S. Bouwman, Hans J. M. Swarts, Annemarie Oosting, Jaap Keijer, Evert M. van Schothorst

**Affiliations:** 1 Human and Animal Physiology, Wageningen University, De Elst 1, Wageningen 6708 WD, The Netherlands; jose.fernandezcalleja@wur.nl (J.M.S.F.-C.); lianne.bouwman@wur.nl (L.M.S.B.); hans.swarts@wur.nl (H.J.M.S.); jaap.keijer@wur.nl (J.K.); 2 Danone Nutricia Research, Uppsalalaan 12, Utrecht 3584 CT, The Netherlands; annemarie.oosting@danone.com

**Keywords:** glycemic index, nutrition, amylose, amylopectin, carbohydrates, C57BL mice, sexual dimorphism, indirect calorimetry, adipose tissue, metabolic flexibility

## Abstract

Starches of low and high digestibility have different metabolic effects. Here, we examined whether this gives differential metabolic programming when fed in the immediate post-weaning period. Chow-fed mice were time-mated, and their nests were standardized and cross-fostered at postnatal days 1–2. After postnatal week (PW) 3, individually housed female and male offspring were switched to a lowly-digestible (LDD) or highly-digestible starch diet (HDD) for three weeks. All of the mice received the same high-fat diet (HFD) for nine weeks thereafter. Energy and substrate metabolism and carbohydrate fermentation were studied at the end of the HDD/LDD and HFD periods by extended indirect calorimetry. Glucose tolerance (PW 11) and metabolic flexibility (PW14) were analyzed. Directly in response to the LDD versus the HDD, females showed smaller adipocytes with less crown-like structures in gonadal white adipose tissue, while males had a lower fat mass and higher whole body fat oxidation levels. Both LDD-fed females and males showed an enlarged intestinal tract. Although most of the phenotypical differences disappeared in adulthood in both sexes, females exposed to LDD versus HDD in the early post-weaning period showed improved metabolic flexibility in adulthood. Cumulatively, these results suggest that the type of starch introduced after weaning could, at least in females, program later-life health.

## 1. Introduction

Early life experiences in critical periods during prenatal and postnatal development have the potential to program metabolic health later in life. While early-life nutrition has been identified as a major environmental condition inducing long-lasting effects in the organism, the optimal diet to promote a healthy life from conception to adulthood is still ill-defined. Much emphasis has been placed on nutritional interventions prenatally and during infancy, since this is considered the period of maximal developmental plasticity. However, it is recognized that the critical development period extends after infancy in some organs and systems [1].

Early life exposure to different qualities and quantities of protein and lipids has been shown to have a lasting impact on adult metabolic health [2,3,4,5]. Dietary carbohydrates may also have a role in programming of later-life metabolic health, as both quality and quantity could provide cues for disease development, treatment, and management. A high intake of low glycemic index (GI) foods is associated with improved health outcomes in both adults and children [6,7]. Using highly defined diets, with only the type of starch being different, we have previously shown that a low versus high GI diet delayed obesity-associated disease development in adult mice [8]. Moreover, the low versus high GI diet induced intestinal microbiota hydrogen production in young and adult mice [9]. Thus, the digestibility of starches provides them with different nutritional properties for both the host and the intestinal microbiota through fermentation [10].

The introduction of solid foods to gradually replace breast milk—or weaning—is a crucial period in the life course. In humans, this period also represents the transition from a high-fat to a high-carbohydrate content in the diet [11]. Importantly, it is during this transition that decisive interactions between the organism and the gut microbiota are being established [12]. Current evidence-based recommendations for complementary feeding are mainly focused on the time of introduction of allergenic foods and solids, with particular attention to protein and fat [13]. However, the rationale for choosing carbohydrates in complementary foods is only based on the development of taste preferences and the prevention of caries [13]. Clearly, carbohydrate intake during early life should also be examined from a metabolic health perspective [14]. 

The recommendations of the joint Food and Agriculture Organization of the United Nations and the World Health Organization (FAO/WHO) expert consultation for carbohydrate intake are virtually the same for all individuals over two years of age [15], and did not substantially change over the course of 10 years due to limited new data. Rodent models are instrumental in developmental programming research due to short gestation and maturity periods and the possibility of exploring molecular mechanisms in ways that would be impractical or unethical in humans [16]. 

The strongest evidence for programming by early-life carbohydrates has been obtained from studies in rats fed a high-carbohydrate milk formula during the suckling period [17]. Another stepping stone in this area is the work of Gugusheff et al. [18], which suggested that starches of different digestibility consumed by dams during the perinatal period as well as their offspring until early adulthood had long-term consequences for metabolic health. However, due to the study design, it was impossible to distinguish between the direct effects and metabolic programming effects, as offspring consumed these intervention diets until the end of the study. 

Several rodent studies incorporating the post-weaning period of growth and development into the programming model have demonstrated metabolic programming by dietary lipids [19,20], protein [21], calcium [22], and the fat:carbohydrate ratio [23], and a lack of differential programming effects in the case of glucose versus fructose [24]. 

In this study, we investigated the long-term effects of starches of different digestibility consumed only during the specific window from weaning until mid-adolescence, on adult metabolic health. We focused on the general aspects of the resulting phenotypes, with an emphasis on metabolic function, e.g., body composition development and whole body metabolism, in both female and male mice. We hypothesized that a lowly-digestible starch post-weaning diet would be protective against the metabolic impairment induced by a high-fat diet during adulthood. We concluded that the early post-weaning period is indeed amenable to metabolic programming by dietary starches, since females consuming a lowly-digestible versus highly-digestible starch diet in the early post-weaning period had a better metabolic flexibility in adulthood. 

## 2. Materials and Methods

### 2.1. Animal Model

**Figure 1:**
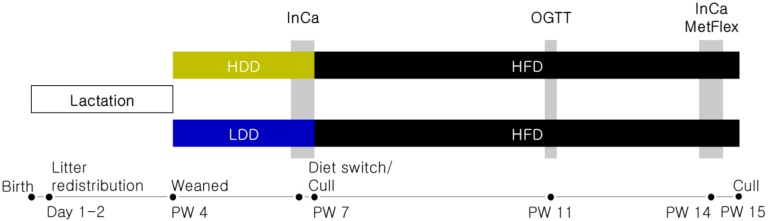
Experimental design. Female and male mice fed standard rodent chow were time-mated and their offspring were redistributed at postnatal day 1–2 to standardized nests. At the end of postnatal week (PW) 3, after weaning, the pups were individually housed and fed either a highly-digestible starch diet (HDD) or a lowly-digestible starch diet (LDD). In PW 6, all of the mice had their basal metabolic phenotype assessed by indirect calorimetry (InCa). A subgroup of animals of each diet and sex was dissected at the end of PW 6 to further assess the direct effects of the post-weaning dietary intervention. The remaining mice were switched to a high-fat diet (HFD) from PW 7 onwards to study metabolic programming in an obesogenic environment. In PW 11, mice underwent an oral glucose tolerance test (OGTT). At the end of HFD-feeding, basal metabolism was measured, and metabolic flexibility (MetFlex) was assessed by InCa. Mice were culled at the end of PW 15, and their blood and tissues were harvested for further analysis.

The study was approved by the Animal Experiment Committee of Wageningen University (DEC 2014085) and performed in accordance to European Union (EU) directives 86/609/EEC and 2010/63/EU. All of the mice (C57BL/6JRccHsd; Harlan Laboratories BV, Horst, The Netherlands) were housed in polycarbonate type II cages enriched with wood chips and wood shavings, with free access to drinking water and food, at 23 ± 1 °C, 50 ± 5% humidity, on a 12-h light/dark cycle. A schematic overview of the study design is shown in Figure 1. Female and male mice (17–19 weeks old) were fed standard rodent chow (26% w/w protein, 38.8% w/w starch, 4.6% w/w sugar, 6.5% w/w fat; AM-II, AB Diets, Woerden, The Netherlands) and time-mated. At postnatal days 1–2, offspring were redistributed across foster dams to produce standardized litters of six pups and a sex ratio of 3:3 or 4:2. At the end of postnatal week (PW 3), all of the mice were housed individually and assigned either a highly-digestible starch diet (HDD; Research Diet Services, Wijk bij Duurstede, The Netherlands; details are described below in Section 2.2) or a lowly-digestible starch diet (LDD; Research Diet Services) stratified according to body weight (BW; *n* = 24 per sex and diet; one male on LDD was excluded due to incisor malocclusion). At the end of PW 6, a subgroup of mice of each sex and dietary group was sacrificed and white adipose tissue (WAT) from gonadal and mesenteric origin, liver, pancreas, and intestine and its contents, were dissected, snap-frozen in liquid nitrogen, and stored at −80 °C until further analysis. The remaining mice (*n* = 12 per sex and diet) were switched to a high-fat diet (HFD, Research Diet Services BV) and continued on this diet until sacrifice in PW 15. Food intake (FI) was determined weekly. BW and body composition (BC; EchoMRI 100V, EchoMedical Systems, Houston, TX, USA) were determined weekly from PW 4–6 and biweekly from PW 7–15. Two indirect calorimetry (InCa) measurements (PW 6 and PW 14) and an oral glucose tolerance test (OGTT; PW 11) were carried out as described below (Section 2.3 and Section 2.4, respectively).

### 2.2. Experimental Diets

All of the experimental diets were based on the BIOCLAIMS standard diet [25]. Both HDD and LDD contained 20 energy percentage (en%) protein, 55 en% carbohydrates, and 25 en% fat, with highly-digestible or lowly-digestible starches as the sole difference and source of available carbohydrate (Cargill, Sas van Gent, The Netherlands; incorporated into pelleted diets by Research Diet Services), as published [9]. The HFD contained 20 en% protein, 40 en% carbohydrates, and 40 en% fat [26]. Detailed diet formulations are shown in Table 1.

### 2.3. Oral Glucose Tolerance Test

An OGTT was performed five hours after food withdrawal in PW 11 by the administration of glucose (2 g kg^−1^ BW) by oral gavage as published [24]. 

### 2.4. Indirect Calorimetry (InCa) and Metabolic Flexibility

The general procedure for indirect calorimetry measurements has been described previously [24], with minor adjustments. After an 18-h adaptation period, the energy expenditure (EE), respiratory exchange ratio (RER), locomotor activity, and food intake were measured in a PhenoMaster indirect calorimetry system (TSE Systems GmbH, Bad Homburg, Germany), which was extended with hydrogen (H_2_) and methane sensors for real-time measurements of intestinal microbial fermentation [9]. Uncorrected EE values were used, since lean mass (LM) was not significantly different between dietary groups directly before or after each InCa period. To assess metabolic flexibility (PW 14), mice were fed a restricted amount of HFD (1.1 g, which is equivalent to about 55% of average food intake during the dark phase) two hours prior to the dark phase to induce a fasting state the next morning. Approximately one hour before the following dark phase, all of the mice were given a meal challenge (HDD) *ad libitum*, and measurements continued until the following light phase. The switch from predominantly fat oxidation (RER = 0.7) toward net carbohydrate oxidation (RER = 1.0) upon refeeding was used as a measure of metabolic flexibility [27]. A selection of data obtained from animals at PW 6 has been previously reported: EE, RER, and H_2_ production [9]. 

### 2.5. Sacrifice

At the end of PW 6 and PW 15, mice were deprived of food at the onset of the light phase and decapitated two to six hours thereafter. Blood glucose was measured in duplicate with a Freestyle glucose meter (Abbott Diabetes Care, Hoofddorp, The Netherlands). Whole blood was collected in chilled MiniCollect serum tubes (Greiner Bio-One BV, Alphen aan de Rijn, The Netherlands), spun down at 4 °C for 10 min at 3000× *g*, and the resulting serum aliquoted and stored at −80 °C. Liver, mesenteric white adipose tissue (mWAT), and pancreas were weighed and snap-frozen in liquid nitrogen. A ~2 g clip was attached to the distal end of the small intestine and hung next to a ruler to determine the length of the small intestine. Thereafter, the small intestine and colon were each cut longitudinally, rinsed in ice-cold RNase-free phosphate-buffered saline to remove their contents, and weighed separately. Caecum contents were extracted, weighed, and snap-frozen. One pad of gonadal white adipose tissue (gWAT) was snap-frozen; the other pad was weighed, fixated in 4% paraformaldehyde overnight, and embedded in paraffin. Samples were stored at −80 °C until further analysis. 

### 2.6. Serum Measurements

Serum levels of insulin, leptin, and adiponectin were determined as described using commercial kits [24].

### 2.7. Hepatic Triglycerides and Glycogen Content

Hepatic triglycerides were determined using a commercial kit as described [24]. Part of the same liver lobe was used for glycogen determination based on published protocol [28] with the following minor adaptations: protein-free and lipid-free extracts were obtained by homogenization of ~100 mg of liver tissue in cold 7% HClO_4_, centrifugation at 4 °C for 15 min at 1500× *g*, and further extraction with petroleum ether. Glycogen concentration in the extracts was determined in triplicate by adding iodine–iodide solution in the presence of CaCl_2_ (260 µL of reagent added to 10 µL of sample), and measuring absorbance at 460 nm.

### 2.8. Gonadal White Adipose Tissue (gWAT) Histology

The paraffin-embedded gWAT pads of six mice per experimental group were selected to represent the average fat mass (FM) and gWAT weight of the complete group. Paraffin blocks were cut into 5-µm thick slices with 150-µm separation in between sections to ensure different areas within the tissue could be studied. Four to five sections per animal were used to determine the adipocyte area by hematoxylin–eosin (HE) staining, and the number of macrophages and crown-like structures (CLS, MAC-2 staining), as published [26,29]. All of the parameters were based on 1000 intact adipocytes per animal. The fluorescence of eosin resulting from HE staining was used to visualize adipocytes with a Leica DM6B microscope equipped with a DFC365FX camera (Leica Microsystems, Wetzlar, Germany), and fluorescent photographs were analyzed using CellProfiler software v. 2.1.1 using the adipocyte pipeline by the Rodeheffer Laboratory to measure cell area [30,31]. Adipocyte diameter was calculated from its area based on a circular shape. 

### 2.9. Quantitative Real-Time Reverse-Transcription Polymerase Chain Reaction (RT-qPCR)

Total RNA was isolated from gWAT using TRIzol reagent (Invitrogen, Breda, The Netherlands) as described [32], and cDNA was synthesized with the iScript cDNA synthesis kit (Bio-Rad Laboratories, Veenendaal, The Netherlands). The expressions of genes involved in macrophage infiltration (chemokine (C–C motif) ligand 2, *Ccl2*; lectin, galactose binding, soluble 3, *Lgals3*; S100 calcium binding protein A8, *S100a8*), insulin signaling (insulin receptor substrate 2, *Irs2*), and lipid metabolism (fatty acid binding protein 4, *Fabp4*) were analyzed in duplicate by RT-qPCR with iQ SYBR Green Supermix (Bio-Rad). Primers were designed to span exon–exon junctions to prevent the amplification of genomic DNA using the NCBI Primer BLAST tool, and PCR products were run on a gel to confirm amplicon sizes when necessary. Standard curves were constructed with cDNA pooled from all samples, a control containing no cDNA, a negative RT control, and a melt curve at the end of the each run, and included for quality control. In the case of the lowly-expressed *Ccl2*, *Lgals3*, and *S100a8* transcripts, cDNA was pre-amplified for 10 cycles with SsoAdvanced PreAmp Supermix (Bio-Rad) and the corresponding primers, according to the manufacturer’s instructions. Full details of all of the primers can be found in Table S1. Normalized gene expression levels were computed with CFX Manager software, v. 3.1. (Bio-Rad) and used for statistical comparisons. 

### 2.10. Data Analysis

Statistical analyses were performed in GraphPad Prism 5.04 (GraphPad, San Diego, CA, USA), and female and male data were analyzed separately. Data was tested using the D’Agostino and Pearson omnibus for normality. Non-normally distributed data was log-transformed and re-tested for normality. Two-tailed comparisons between two groups were made using unpaired Student’s *t*-tests or Mann–Whitney *U*-tests for normally and non-normally distributed data, respectively. Other group comparisons were tested with two-way ANOVA (adipocyte size frequency distribution and macrophage infiltration), with repeated measurements for matched time course data (OGTT, RER, and carbohydrate intake during InCa) and Bonferroni’s post hoc test. Correlations analyses were performed using Pearson correlation on normally distributed data and Spearman correlation for non-normally distributed data. The incremental area under the curve (iAUC) of glucose during OGTT was also calculated in Prism. Statistical significance was set at *p* < 0.05 for all of the comparisons.

## 3. Results

### 3.1. Direct and Long-Term Effects on Body Weight and Body Composition by Post-Weaning Starches

**Figure 2:**
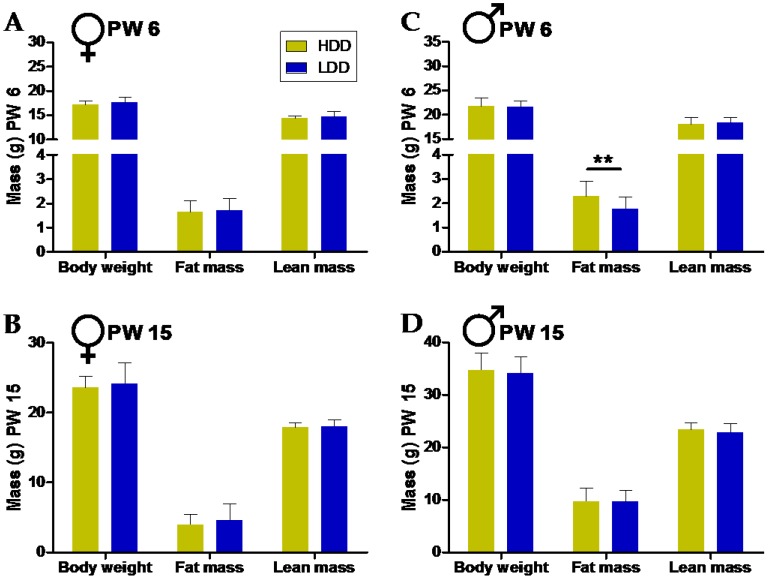
Direct and long-term effects of the type of starch consumed in the early post-weaning period on body weight (BW) and body composition. BW, fat mass (FM), and lean mass (LM) of females directly after exposure to HDD or LDD (**A**; PW 6, *n* = 24 per group), and after nine weeks on a HFD (**B**; PW 15, *n* = 12 per group). The BW, FM, and LM of males after exposure to HDD or LDD (**C**; PW 6, *n* = 24 for HDD and *n* = 23 for LDD), and after nine weeks on a HFD (**D**; PW 15, *n* = 12 per group). Note truncated x-axis in panels **A** and **C** to enhance visualization. Data shown as mean ± standard deviation (s.d.). Statistical differences denoted as ** *p* ≤ 0.01.

Directly after three weeks of consumption of HDD or LDD (PW 4–6), there was no difference in body weight or lean mass between the two groups (Figure 2A,C; Figure S1A,C,D,F). However, males on the HDD developed more fat mass compared to those fed the LDD in this period (Figure 2C; Figure S1E). This was not seen in female mice (Figure 2A; Figure S1B).

Following the intervention period, all of the mice received nine weeks of HFD feeding. At 15 weeks of age, there were no significant differences seen in body weight or body composition in neither females nor males (Figure 2B,D). 

### 3.2. Direct and Long-Term Effects on Basal Metabolism

Energy expenditure and locomotor activity were not affected by the type of starch neither at the end of the intervention nor upon HFD feeding, for both females and males (Table 2). However, males consuming the LDD showed a lower RER compared to males fed the HDD (Table 2), indicating increased fat over carbohydrate oxidation. This difference in substrate utilization was completely absent in the females. The effects on basal RER that were seen in males disappeared, and thus were not metabolically programmed at the end of the HFD period (Table 2). This cumulatively suggests that basal metabolism, including fuel utilization, is not programmed by the type of starch consumed in the early post-weaning period either in females or males. 

A novel parameter that can be measured using our extended indirect calorimetry system is production of the fermentation gases hydrogen (H_2_) and methane [9]. H_2_ is exclusively formed by gut microbes as a product of carbohydrate fermentation [33], and as such represents a convenient marker for gut microbiota activity. In line with the known differences in digestibility of 40% amylose and 60% amylopectin compared with 100% amylopectin within the food matrix in vitro and in vivo [9], there were significant differences in H_2_ output between mice consuming HDD or LDD in both females and males, with LDD mice producing approximately eight times more H_2_ than HDD mice over 24 h (Table 2, [9]). H_2_ production was relatively low and similar for all of the mice consuming the HFD (Table 2). At the same time, absolute methane levels being at ambient levels indicated an absence of methane production at both the end of the intervention and HFD-feeding periods in any group.

### 3.3. Direct and Long-Term Effects on Other Physiological Parameters at Sacrifice

There were no obvious differences in the weight of metabolic organs and circulating parameters after the early post-weaning intervention or at the end of the HFD period (Table 3). An important exception was the elevated serum leptin concentrations in HDD males in PW 6, which is consistent with the increased fat mass. There were significant differences in the gross energy intake during HDD and LDD feeding, with mice cumulatively ingesting about 0.1 MJ more on the LDD (Table 3); however, this did not lead to a significant difference in body weight or lean mass (Figure 2A,C).

The most remarkable finding in the overall phenotype at sacrifice was the direct effect of the type of starch on intestinal tract morphology. Both the weight and length of the small intestine, and colon weight were increased in females and males consuming the LDD (Table 3). In addition, despite being in the post-absorptive state, cecum contents were increased in females and males fed the LDD (Table 3). At the end of the HFD period, these differences were normalized to values similar to the LDD-fed mice in early life (Table 3).

### 3.4. Long-Term Effects on Glucose Tolerance

**Figure 3:**
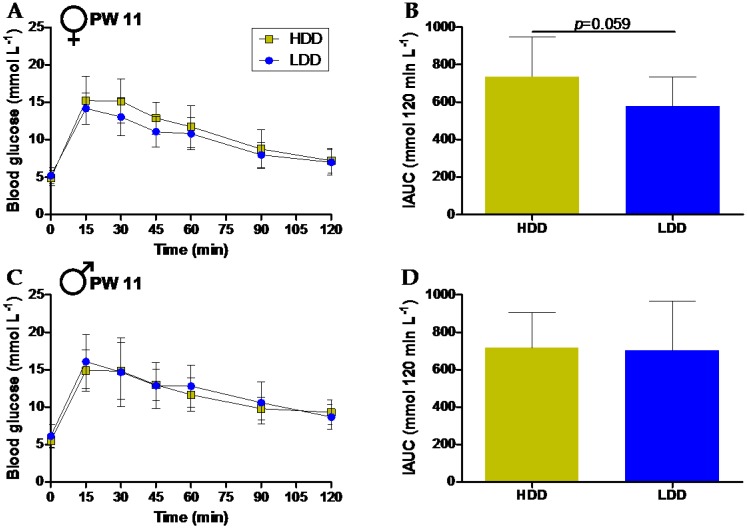
Glucose tolerance after five weeks of HFD feeding (PW 11). Plasma glucose concentrations measured directly before (0 min) and after oral administration of a glucose bolus (2 g kg^−1^ BW) in female (**A**, *n* = 12 for HDD and *n* = 11 for LDD) and male (**C**, *n* = 11 for HDD and *n* = 12 for LDD) mice. Incremental area under the curve (iAUC) for blood glucose over the 120min period for females (**B**) and males (**D**). Data shown as mean ± s.d.

The perturbation of homeostasis may allow the detection of subtle or early differences in metabolic phenotypes, particularly those induced by nutritional interventions [34]. We first employed an OGTT to challenge glucose homeostasis in PW 11, when mice had been consuming a HFD for five weeks. No differences were seen at baseline in glycemia (Figure 3A,C) or insulinemia (females: 0.7 ± 0.1 ng mL^−1^ versus 0.7 ± 0.2 ng mL^−1^; males: 1.6 ± 0.3 ng mL^−1^ versus 1.5 ± 0.4 ng mL^−1^; mean ± s.d., HDD and LDD respectively, *n* = 12 per group). The glycemic response to the glucose bolus was also similar between groups, with only a trend for a lower iAUC in the LDD females (Figure 3B,D).

### 3.5. Long-Term Effects on Metabolic Flexibility

**Figure 4:**
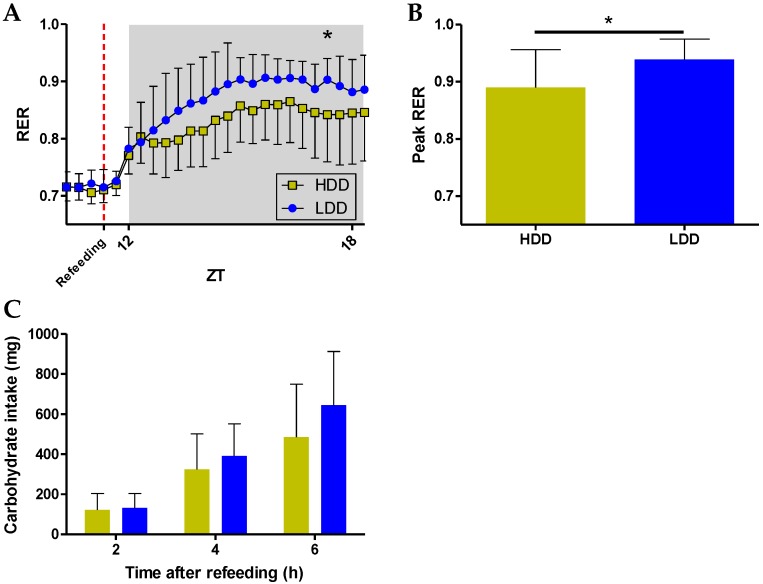
Metabolic flexibility of female mice after eight weeks of HFD feeding (PW 14). (**A**) RER evolution one hour before refeeding until seven hours upon *ad libitum* refeeding with a high carbohydrate diet (HDD). Statistical comparison was performed on all of the data points from the moment of food restriction (additional data points not shown to enhance visualization). (**B**) Mean peak RER values achieved within seven hours after refeeding. (**C**) Cumulative carbohydrate intake calculated from the automatic records of food intake after access to the refeeding diet. *n* = 13 for HDD and *n* = 11 for LDD. Data shown as mean ± s.d. Statistical difference denoted as * *p* ≤ 0.05.

In contrast to the OGTT, which focuses on glucose metabolism, we also performed a nutritional challenge test that can impact a larger array of metabolic processes [35], which has been previously used for the detection of nutritionally-induced metabolic differences [26,36]. A fasting–refeeding challenge was performed in indirect calorimetry after eight weeks of HFD feeding (PW 14), using the HDD as the *ad libitum* refeeding meal. The diet provides a rapid influx of glucose into the bloodstream, competing with protein and fat as additional substrates. The highly coordinated response of the organism to switch from predominantly fat oxidation (low RER) to glucose oxidation (high RER) was quantified as an indicator of metabolic flexibility [27,36]. Since the potential programming of glucose metabolism was hinted at only in females (Figure 3), we next focused primarily on females, presenting male data when available. The decline in RER after food restriction evolved in a virtually identical manner between mice fed HDD or LDD in the early post-weaning period, for both females and males, which also ensured that all of the mice were equally fasted before regaining access to food. Upon refeeding and particularly after the first hour of refeeding, the RER in females followed different trajectories, with a significant interaction between time and the post-weaning diet (*p* < 0.0001, Figure 4A). LDD females constantly had a numerically higher RER and reached a statistically significant higher RER at about six hours after access to food (Figure 4A). Similarly, the peak RER values that were reached within the refeeding period were higher in the LDD females (Figure 4B). The response in both the HDD and LDD males within three hours of refeeding was similar to that of the HDD females (Figure S2A), and the peak RER values that were reached upon refeeding were not significantly different between the male groups (Figure S2B).

To corroborate equal food intake during the challenge, we analyzed automatic food intake following access to food. There were no statistical differences in food intake between groups, neither in females (Figure 4C) nor in males (Figure S2C). Thus, our data points toward an improved capacity of LDD females to adapt fuel utilization to fuel availability, i.e., a better metabolic flexibility. 

### 3.6. Direct and Long-Term Effets on Adipose Tissue in Females

**Figure 5:**
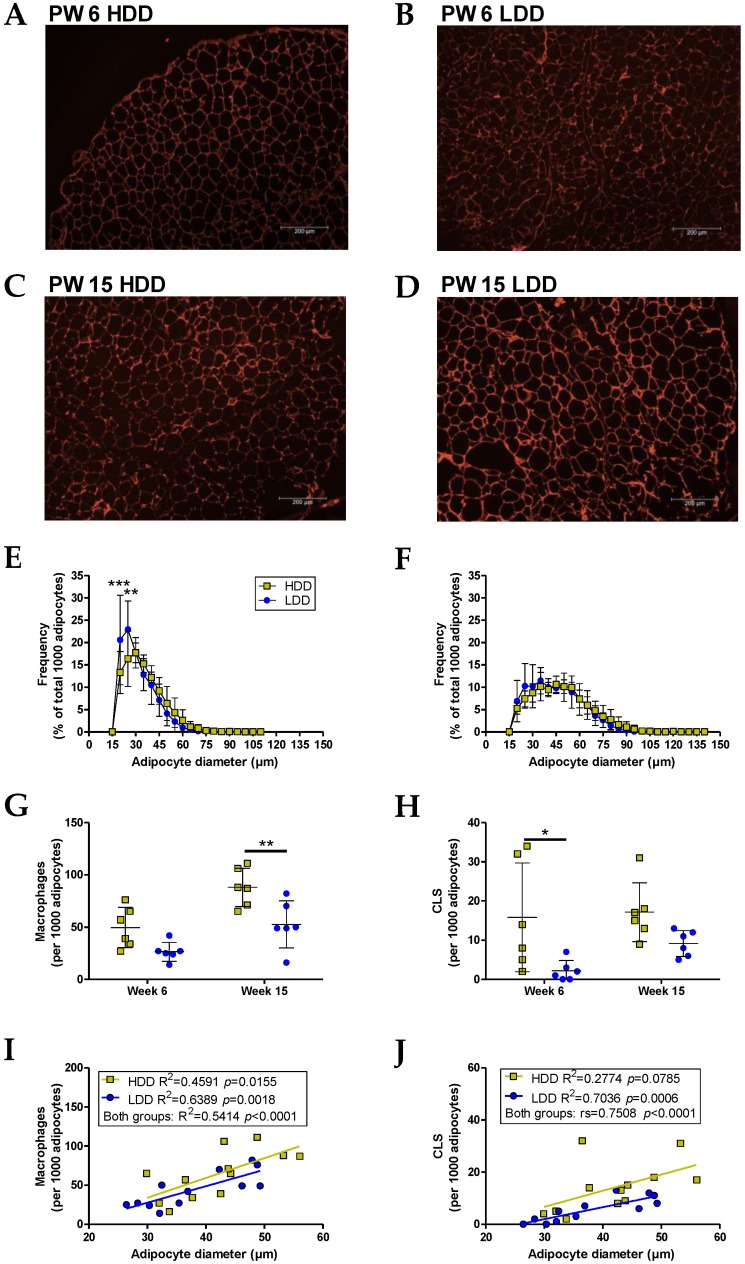
Adipose tissue histology, adipocyte size distribution, and macrophage infiltration markers in the gWAT of females at the end of the post-weaning intervention (PW 6) and after the HFD feeding period (PW 15). Representative histological pictures of gWAT using hematoxylin–eosin (HE) staining in HDD-fed (**A**) and LDD-fed mice (**B**) at the end of the post-weaning intervention (PW 6), and HDD-fed (**C**) and LDD-fed mice (**D**) after the HFD feeding period (PW 15). Photos were made using fluorescent microscopy. Distribution of adipocyte diameter (5 µm bins) in gWAT pads collected in PW 6 (**E**) and PW 15 (**F**). Total count of macrophages (**G**) and crown-like structures (CLS, **H**) identified in the same areas using MAC-2 immunohistochemical staining. Correlation plots of adipocyte diameters with (**I**) macrophage and (**J**) CLS counts for all of the animals in each group. All of the coefficients were obtained with Pearson correlation except for the CLS of the combined HDD and LDD groups (rs = Spearman correlation coefficient). *n* = 12 per dietary group, *n* = six per age (PW 6 or PW 15). Data shown as mean ± s.d. Statistical differences denoted as ** *p* ≤ 0.01 and *** *p* ≤ 0.001.

While mean adipocyte size in gonadal white adipose tissue (gWAT) was not different between groups (PW 6: 35.4 ± 4.5 µm versus 31.6 ± 4.0 µm; PW 15: 48.2 ± 5.4 µm versus 44.5 ± 6.4 µm; mean ± s.d., HDD and LDD respectively, representative pictures in Figure 5A–D), the distribution of adipocyte diameter in mice directly exposed to LDD was shifted toward smaller adipocytes compared to the HDD intervention (interaction between post-weaning diet and diameter bin: *p* = 0.0016, Figure 5E). Nonetheless, the statistical differences in adipocyte size distribution disappeared after nine weeks of HFD feeding, although they were still bearing some visual resemblance to the distribution at PW 6 (Figure 5F). 

Adipocyte size has been linked to macrophage infiltration and inflammation [29], which in turn has been linked to metabolic flexibility [37]. We therefore characterized macrophage and CLS abundance in gWAT depots. Both directly after the early post-weaning intervention and at the end of the HFD period, the gWAT pads of HDD mice harbored higher numbers of macrophages and CLS; however, only CLS in PW 6 and macrophages in PW 15 achieved statistical significance (Figure 5G,H). Interestingly, the mRNA levels in the gWAT pads in PW 15 of three macrophage markers, *Lgals3*, *Ccl2*, and *S1008a*—the latter a key gene associated with M1 macrophages—as well as two other genes linked to adipose tissue function (*Irs2* and *Fabp4*), revealed no significant differences between groups (Figure S3). On the other hand, the histological data was consistent with the long-established association of macrophage abundance and CLS formation with adipocyte size in rodents [29,38] (Figure 5I,J). Collectively, this data indicates that the type of starch had clear direct effects on adipose tissue morphology in females with associated differences in inflammation markers. These differences did not persist later in life.

## 4. Discussion

The direct differential effects of lowly-digestible versus highly-digestible-starch diets were seen in females for gWAT morphology and CLS abundance, and in males for whole body substrate metabolism and fat mass gain, with robust effects in the gut physiology in both sexes. Female mice that were subsequently fed a HFD into adulthood showed an improved capacity to adapt energy substrate utilization to substrate availability at the whole body level; however, this effect was not seen in males. This shows that metabolic flexibility in later life can be programmed by the type of starch in the early post-weaning diet in a sex-dependent manner.

The direct metabolic effects by differences in starch digestibility can be due to two main factors: postprandial glycemia and gut microbiota. A highly-digestible starch will be more readily absorbed in the small intestine and stimulate insulin secretion more pronouncedly than a lowly-digestible starch, whereas a fraction of lowly-digestible starch will reach the cecum and colon and interact with gut microbiota. Both hyperglycemia and hyperinsulinemia could independently explain adipose tissue macrophage (ATM) homing and adipose tissue inflammation [39]. At the same time, gut microbiota is able to influence host health through multiple mechanisms [40]. Short-chain fatty acids (SCFA) derived from microbial fermentation have anti-inflammatory and other properties, directly inhibiting lipid storage via free fatty acid receptor 2 and indirectly increasing glucose uptake in adipocytes via the insulin-reinforcing action of gut peptide YY (PYY) [41]. The inhibition of fat storage and increased glucose disposal to adipose tissue would promote fatty acid utilization in other tissues, which is consistent with the lower RER seen in males consuming LDD. Moreover, as Zeevi et al. [42] demonstrated, postprandial glycemic responses to the same meal depend partly on microbiota features. This, together with the observational evidence that a microbiota composition with a higher capacity for carbohydrate fermentation dampens weight loss in obese adults [43], suggests a particularly important interaction between the two main factors that are involved in our study (postprandial glycemia and gut microbiota). Interestingly, our post-weaning LDD intervention led to a dramatically different fecal microbiota composition in both sexes versus HDD-fed mice, with the increase in *Parasutterella*, *Bacteroides*, and *Alloprevotella* abundance after three weeks of LDD feeding strongly correlating only with H_2_ production, but not with body weight, fat mass, or food intake [9]. It is most likely that a combination of host and microbiota-mediated mechanisms explains the direct phenotypes of HDD and LDD mice. Although it has been demonstrated that some of the metabolic effects of resistant starches appear to be independent of the presence of a gut microbiota [44], the starch in our LDD cannot quite be considered a resistant starch per se [45]. 

The observed direct impact of starch digestibility on metabolic health in the present study is in accordance with recent findings on dietary GI-induced effects in rodent models. Particularly, the increased fat mass in males fed a HDD is consistent with a recent meta-analysis of murine GI studies showing that males benefit more from a low GI diet compared to females for several metabolic outcomes, including adiposity [46]. The lower RER observed in LDD males is also consistent with previous studies in rodents [47]. Our data do not show effects on BW and fasting glycemia, which may be due to the duration of dietary exposure [46]. A key observation in this study is the sex-specific effect of the type of starch on substrate utilization. This merits further investigation, especially because of the lack of experimental work in female models of nutrition, including starches of different digestibility [46]. It is unclear whether these differences are due to the effects of circulating sex hormones, which have been shown to be able to protect females from HFD-induced obesity and inflammation [48], or alternatively, to developmental differences in metabolic regulation [49]. 

Perhaps the strongest direct exposure effects were the enlargement of the lower intestinal tract in the LDD-fed female and male mice, supporting previous data on starches of low digestibility [18,50]. This enhanced growth of the intestine is in line with the trophic effects of SCFA [51]. Indeed, we observed increased SCFA in the cecum and colon upon LDD versus HDD feeding, as well as increased H_2_ production [9]. Yet another possibility is an effect of energy dilution due to the lower digestibility of the LDD. It has been demonstrated that mice invest in the growth of the stomach, ileum, cecum, and colon over three months of calorie restriction, at the same time preferentially utilizing WAT depots [52]. This investment in the alimentary tract was associated with a parallel increase in the assimilation efficiency of the diet [52]. In light of this evidence, it might be speculated that gut microbiota and host interact to maximize energy harvest in response to the lower nutrient availability in the LDD.

The observed acute responses could have potential lifelong programming consequences. Indeed, after a nine-week period of the HFD, females fed the LDD showed a better metabolic flexibility in adulthood, supporting the potential of the early post-weaning diet to program metabolic health. In this sense, it is somewhat surprising that most of the other phenotypic parameters that were measured were similar, irrespective of the early post-weaning diet, and existing differences disappeared. Even the strong effects of the type of starch on the intestinal parameters seen in both sexes appeared to be absent later in life. 

Sexual dimorphism in animal models of metabolic programming has been repeatedly observed, not only upon prenatal, but also postnatal exposure [53]. With regard to dietary carbohydrates, together with the study of Gugusheff et al. [18], our work provides evidence that females are more susceptible to the long-term effects of particular types of starch on metabolic health. This is interesting given that, in line with our data shown here, the direct effects of starch digestibility are only seen in male rodents [46]. Surprisingly, males seemed largely unaffected over the long term, although we cannot exclude the possibility of programming other physiological outcomes. Moreover, sexual dimorphism in response to fasting has been only recently understood in great detail, with females actively promoting lipogenesis from amino acids, and males generally toning down anabolic pathways [54], which could have major implications for metabolic flexibility. Additional studies would be needed to clarify whether females and males have indeed different developmental windows that are amenable to metabolic programming by starches.

On the whole, the metabolic consequences of early post-weaning starches were mild. This might be due to the physiologically relevant dietary levels of starches during the intervention, as well as the fat content during the period thereafter. Programming effects may have been more apparent using e.g., a 60 en% fat HFD, which was required to reach maximal body weight and adiposity in a recent ‘wild-type’ mouse study with 29 diets varying in macronutrient proportions [55]. Alternatively, the mice we used are too healthy, which is supported by the OGTT responses. The usage of relevant disease models, such as mice with an impaired redox homeostasis (C57BL/6J mice from Jackson Laboratory in fact, as they have a mutated non-functional *Nnt* gene, in contrast to C57BL/6JRccHsd strain we used here, which has a functional *Nnt* gene) might provide opportunities. The strongest consequences of early post-weaning starches could also have been delayed, with aging being an important factor in the development of metabolic disease. Nevertheless, within nearly the same time span and study design, beneficial metabolic programming effects have been observed using specific lipids in the early post-weaning diet [56,57]. 

On the question of what mechanisms could be responsible for the long-term effects of starches in the early post-weaning period, epigenetic processes are thought to underlie a considerable amount of programming phenomena, along with changes in tissue structure and accelerated cellular aging [58,59]. Neither we nor others [17] have attempted to unravel epigenetic mechanisms specifically in the context of programming by carbohydrates, although there is a strong possibility that such mechanisms take place. For instance, key components of the machinery governing metabolic flexibility can be programmed by maternal nutrient and protein restriction, and are susceptible to epigenetic changes [60,61,62]. Moreover, some tissues retain plasticity to epigenetic modifications through early adulthood, as is the case for the brain and the colonic mucosa [63,64], with butyrate—a SCFA—being able to cause epigenetic changes in the intestinal epithelium [40]. It is therefore conceivable that highly-digestible and lowly-digestible starches induce cellular biochemical changes that in turn cause epigenetic changes. Exactly which tissues are targeted is unclear.

We see value in placing the early post-weaning window that was chosen in this study within the current evolutionary paradigms of the Developmental Origins of Health and Disease (DOHaD). One of the fundamental premises of the DOHaD framework is that phenotypic adaptations in response to early-life environmental cues, including nutrition, can be predictive of future environments [65]. When the anticipated environment does not match the actual conditions encountered later in life, such early phenotypic responses can become maladaptive and increase disease vulnerability. It is then conceivable that some of the programming effects that we report are not only determined by the post-weaning diet in itself, but also by earlier nutritional cues. In this way, the hormonal and metabolic environment promoted by carbohydrates during the suckling period could be very different to that encountered in the post-weaning period, depending on the carbohydrates introduced. There is at least one report arguing for this kind of carbohydrate mismatch [18]. Human breast milk is considered a low GI food [66], and it also contains indigestible oligosaccharides that can influence the gut microbiome and SCFA profile [67]. Seen in this way, a lowly-digestible starch post-weaning diet could have produced a similar physiological environment as predicted during suckling, whereas a highly-digestible starch diet could fail to match the forecasted conditions and put the organism on course for disease. 

## 5. Conclusions

Although the differences in the programmed adult phenotypes that we observed were subtle, our findings substantiate the notion that vulnerability to an obesogenic environment could partly depend on carbohydrate quality in early life. In line with the view that disease prevention must start with optimal nutrition early in life, our results need to be considered for the post-weaning diets as well as for products that target this period of growth and development. 

## Figures and Tables

**Table 1:** Composition of the experimental diets.

Component	HDD	LDD	HFD
Casein	212.2	212.0	233.5
l-Cysteine	3.0	3.0	3.0
Amylose mix (AmyloGel 03003) ^1^	0.0	568.6	0.0
Amylopectin (C*Gel 04201) ^2^	568.6	0.0	0.0
Wheat starch	0.0	0.0	285.6
Maltodextrin	0.0	0.0	100.0
Glucose	0.0	0.0	70.0
Coconut oil	21.4	21.4	0.0
Sunflower oil	83.1	83.1	0.0
Flaxseed oil	14.2	14.2	4.0
Palm oil	0.0	0.0	206.3
Cholesterol	0.03	0.03	0.097
Cellulose	50.0	50.0	50.0
Mineral mix (AIN-93G-MX)	35.0	35.0	35.0
Vitamin mix (AIN-93-VX)	10.0	10.0	10.0
Choline bitartrate	2.5	2.5	2.5
Total (g)	1000.0	1000.0	1000.0
Gross energy density (kJ g^1^) ^3^	18.9	19.5	20.8
Calculated energy density (kJ g^−1^) ^4^	17.9	17.9	19.8
Protein (energy%)	20.1	20.1	20.0
Carbohydrate (energy%)	54.9	54.9	40.0
Fat (energy%)	25.0	25.0	40.0

All values are in g kg^−1^ unless indicated. ^1^ 60% amylose, 40% amylopectin (Cargill). ^2^ 100% amylopectin (Cargill BV). ^3^ Determined by bomb calorimetry. ^4^ Calculated based on Atwater’s nutritional values. HDD: highly-digestible starch diet; HFD: high-fat diet; LDD: lowly-digestible starch diet.

**Table 2:** Basal indirect calorimetry parameters measured at the end of the post-weaning intervention (PW 6) and subsequently eight weeks into HFD feeding (PW 14).

	Females				Males			
	PW 6		PW 14		PW 6		PW 14	
Parameter	HDD	LDD	HDD	LDD	HDD	LDD	HDD	LDD
EE (24 h, kJ h^−1^)	1.60 ± 0.08	1.65 ± 0.09	1.90 ± 0.12	1.90 ± 0.12	1.80 ± 0.12	1.77 ± 0.11	2.11 ± 0.25	2.15 ± 0.25
EE (LP, kJ h^−1^)	1.46 ± 0.08	1.52 ± 0.11	1.78 ± 0.13	1.79 ± 0.11	1.66 ± 0.13	1.61 ± 0.12	2.01 ± 0.23	2.04 ± 0.24
EE (DP, kJ h^−1^)	1.75 ± 0.09	1.78 ± 0.09	2.03 ± 0.13	2.02 ± 0.14	1.95 ± 0.12	1.93 ± 0.10	2.21 ± 0.27	2.27 ± 0.26
RER (24 h)	0.84 ± 0.04	0.84 ± 0.04	0.86 ± 0.04	0.86 ± 0.04	0.88 ± 0.03	0.85 ± 0.03 ^#^	0.85 ± 0.02	0.85 ± 0.02
RER (LP)	0.81 ± 0.05	0.82 ± 0.05	0.86 ± 0.04	0.86 ± 0.04	0.85 ± 0.03	0.82 ± 0.04 *	0.85 ± 0.03	0.86 ± 0.02
RER (DP)	0.87 ± 0.05	0.87 ± 0.04	0.86 ± 0.05	0.86 ± 0.04	0.91 ± 0.03	0.88 ± 0.02 *	0.84 ± 0.02	0.84 ± 0.02
Activity (24 h, counts × 10^4^)	6.93 (6.13, 7.45)	6.55 (5.88, 6.72)	5.07 (3.89, 7.07)	4.56 (3.85, 6.23)	4.95 (4.13, 6.06)	5.43 (4.43, 5.70)	2.93 (2.31, 3.81)	2.98 (2.37, 3.51)
Activity (LP, counts × 10^4^)	1.46 (1.35, 1.83)	1.46 (1.27, 1.75)	1.36 (1.02, 1.89)	1.03 (0.84, 1.71)	1.4 (1.06, 1.74)	1.10 (0.97, 1.28)	0.80 (0.66, 1.39)	0.84 (0.71, 1.00)
Activity (DP, counts × 10^4^)	5.45 (4.52, 5.57)	4.47 (4.32, 5.06)	3.42 (2.81, 5.24)	3.35 (2.80, 4.73)	3.65 (2.99, 4.28)	4.31 (3.37, 4.33)	1.88 (1.56, 2.50)	2.07 (1.64, 2.54)
H_2_ (24 h, mL)	0.18 (0.14, 0.26)	1.64 (1.14, 2.12) ^§^	0.24 (0.21, 0.32)	0.34 (0.28, 0.39)	0.24 (0.18, 0.35)	1.47 (1.11, 1.90) ^§^	0.46 (0.36, 0.75)	0.38 (0.32, 0.72)
H_2_ (LP, mL)	0.08 (0.06, 0.13)	0.60 (0.46, 0.74) ^§^	0.10 (0.09, 0.16)	0.19 (0.14, 0.20)	0.10 (0.08, 0.14)	0.52 (0.40, 0.61) ^§^	0.21 (0.16, 0.35)	0.18 (0.15, 0.36)
H_2_ (DP, mL)	0.11 (0.07, 0.14)	1.07 (0.66, 1.41) ^§^	0.14 (0.11, 0.17)	0.15 (0.14, 0.20)	0.14 (0.09, 0.21)	0.99 (0.67, 1.32) ^§^	0.25 (0.20, 0.40)	0.21 (0.17, 0.36)

For EE and RER, data is presented as mean ± s.d. For activity and H_2_, data is shown as median (95% CI of mean), since these values often did not follow a normal distribution. Statistically significant differences compared to the HDD for mice of the same age and sex denoted as * *p* ≤ 0.05, ^#^ *p* ≤ 0.01, and ^§^ *p* < 0.0001. DP: dark phase; EE: energy expenditure (averaged per period); LP: light phase; RER: respiratory exchange ratio (average per period); H_2_: hydrogen (cumulative volume produced per period).

**Table 3:** Organ weights and other physiological parameters at the end of the post-weaning intervention (PW 6) and at end of HFD feeding (PW 15).

	Females				Males			
	PW 6		PW 15		PW 6		PW 15	
Parameter	HDD	LDD	HDD	LDD	HDD	LDD	HDD	LDD
Cumulative GE intake (MJ)	0.90 (0.87, 0.91)	1.01 (0.98, 1.03) ^§^	3.36 (3.26, 3.51)	3.24 (3.12, 3.50)	1.00 (0.97, 1.03)	1.13 (1.10, 1.15) ^§^	3.81 (3.61, 4.09)	3.73 (3.58, 4.06)
gWAT (mg)	65 (58, 83)	65 (48, 77)	197 (178, 297)	240 (181, 422)	140 (122, 168)	117 (91, 145)	667 (568, 758)	796 (592, 885)
mWAT (mg)	104 (83, 139)	108 (81, 114)	202 (183, 303)	244 (180, 383)	176 (150, 200)	140 (117, 173)	562 (474, 797)	627 (523, 806)
Liver (g)	0.72 (0.66, 0.77)	0.67 (0.62, 0.75)	0.89 (0.85, 0.95)	0.89 (0.83, 1.01)	1.01 (0.93, 1.06)	1.05 (0.96, 1.11)	1.20 (1.08, 1.52)	1.23 (1.11, 1.49)
Liver (g g^−1^ body weight)	0.042 (0.039, 0.044)	0.039 (0.036, 0.042)	0.039 (0.037, 0.039)	0.039 (0.036, 0.040)	0.048 (0.044, 0.049)	0.049 (0.045, 0.051)	0.036 (0.033, 0.042)	0.036 (0.034, 0.041)
Liver TG (mg g^−1^ wet tissue)	n.m.	n.m.	36.1 ± 13.9	37.6 ± 14.2	n.m.	n.m.	n.m.	n.m.
Liver glycogen (mg g^−1^ wet tissue)	n.m.	n.m.	53.3 ± 24.0	68.7 ± 39.2	n.m.	n.m.	n.m.	n.m.
Pancreas (mg)	235 (200, 262)	255 (228, 296)	322 (299, 347)	319 (281, 380)	261 (254, 287)	282 (248, 303)	383 (344, 534)	363 (326, 489)
Small intestine (cm)	31.9 (30.8, 32.2)	33.8 (32.8, 36.3) ^#^	33.3 (32.5, 33.7)	33.6 (32.9, 34.8)	33.3 (32.8, 34.0)	35.4 (35.0, 37.2) ^§^	35.0 (34.4, 36.8)	35.7 (34.3, 37.4)
Small intestine (g)	0.60 (0.56, 0.61)	0.72 (0.67, 0.84) ^¥^	0.73 (0.69, 0.74)	0.72 (0.68, 0.79)	0.68 (0.65, 0.70)	0.78 (0.74, 0.89) ^§^	0.84 (0.80, 0.94)	0.84 (0.79, 0.94)
Cecum contents (mg)	108 (90, 116)	225 (204, 295) ^§^	104 (91, 125)	108 (87, 143)	152 (121, 162)	273 (250, 359) ^§^	196 (164, 203)	140 (123, 171) *
Colon (mg)	92 (87, 97)	135 (126, 152) ^§^	118 (109, 122)	112 (109, 123)	104 (97, 109)	153 (141, 166) ^§^	139 (126, 151)	136 (129, 159)
Blood glucose (mmol L^−1^)	5.0 ± 0.7	4.7 ± 1.0	4.9 ± 0.5	5.3 ± 0.9	5.8 ± 0.9	6.2 ± 0.7	5.6 ± 0.9	5.8 ± 0.9
Serum insulin (ng mL^−1^)	0.73 (0.56, 0.87)	0.60 (0.48, 0.72)	0.62 (0.58, 1.11)	0.83 (0.65, 1.60)	1.03 (0.90, 1.12)	0.83 (0.72, 1.04)	2.09 (1.71, 3.20)	2.22 (1.93, 2.61)
Serum leptin (ng mL^−1^)	1.6 (1.2, 2.7)	1.3 (0.9, 2.5)	3.9 (2.9, 8,0)	5.0 (2.8, 13.6)	2.9 (2.1, 3.3)	1.8 (1.3, 2.1) ^#^	52.1 (22.2, 125.9)	65.3 (41.6, 101.6)
Serum adiponectin (µg mL^−1^)	n.m.	n.m.	12.3 ± 1.1	12.2 ± 1.5	n.m.	n.m.	n.m.	n.m.

Liver TG, liver glycogen, blood glucose, and serum adiponectin are presented as mean ± s.d. All other data is shown as median (95% CI of mean) since values often did not follow a normal distribution. Statistically significant differences compared to HDD for mice of the same age and sex denoted as * *p* ≤ 0.05, ^#^ *p* ≤ 0.01, ^¥^ *p* ≤ 0.001 and ^§^ *p* < 0.0001. GE: gross energy; gWAT: gonadal white adipose tissue; mWAT: mesenteric white adipose tissue; n.m.: not measured; TG: triglycerides.

## Supplementary Materials

The following are available online at http://www.mdpi.com/2072-6643/10/11/1788/s1, Figure S1: Body weight and body composition development, Figure S2: Metabolic flexibility of male mice after 8 weeks of HFD feeding (PW 14), Figure S3: Gene expression in gWAT of females after the HFD feeding period (PW 15), Table S1: Primers used for gWAT gene expression analysis.
