# Supplementary material for: Direct and Long-Term Metabolic Consequences of Lowly vs. Highly-Digestible Starch in the Early Post-Weaning Diet of Mice
Source: Nutrients. 2018 Nov 17;10(11):1788. doi: 10.3390/nu10111788 (PMC6265974; doi:10.3390/nu10111788)
Supplement: Supplementary file 1 [file nutrients-10-01788-s001.pdf]

Supplementary file

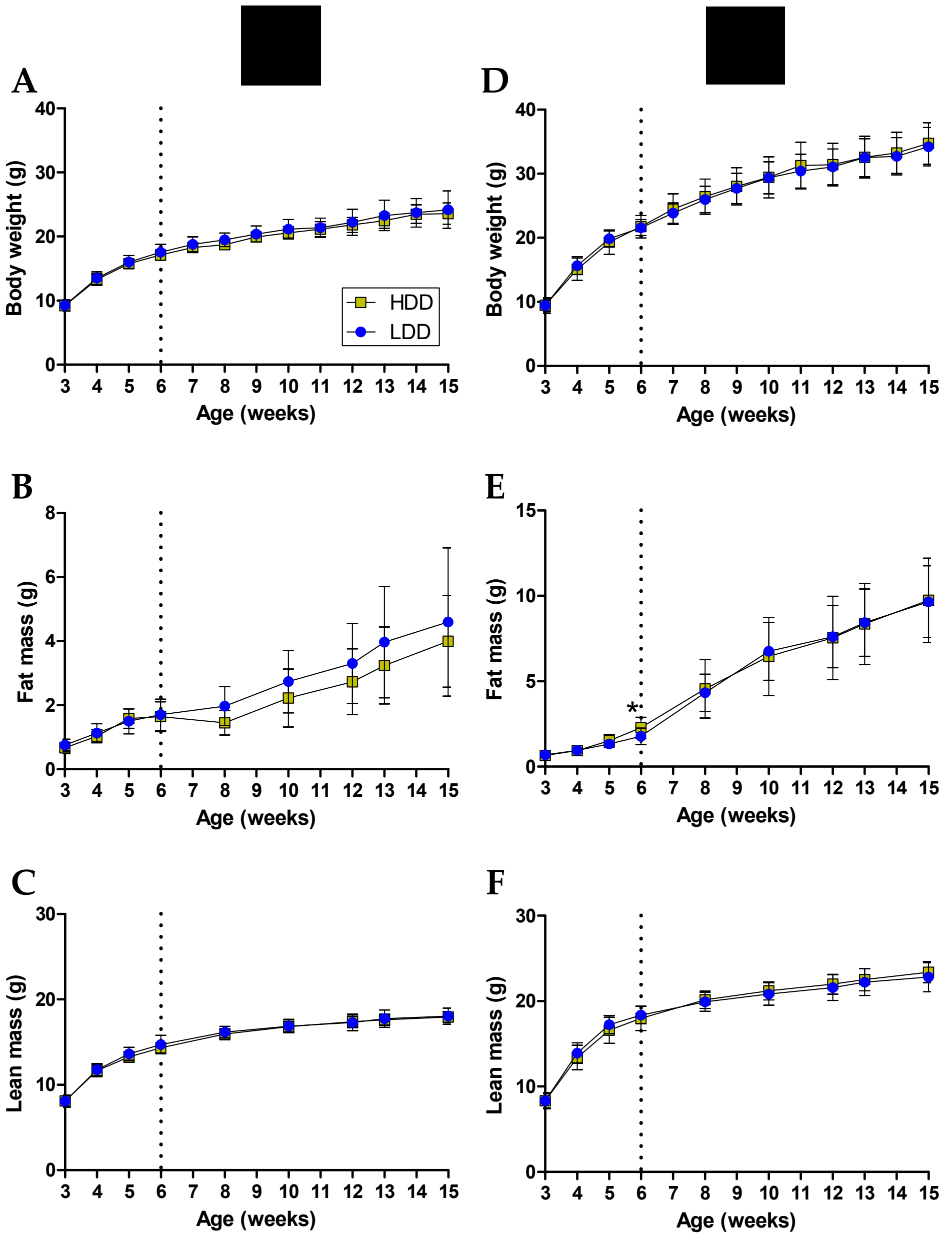

Figure S1. Body weight and body composition development. (**A**) Body weight (BW), (**B**) fat mass (FM), and (**C**) lean mass (LM) of females from weaning at postnatal week (PW) 3 until the end of the study (*n* = 24 per group from PW 3 through 6; *n* = 12 per group from PW 7 through 15). (**D**) BW, (**E**) FM, and (**F**) LM of males from weaning at PW 3 until the end of the study (*n* = 24 for HDD and *n* = 23 for LDD from PW 3 through 6; *n* = 12 per group from PW 7 through 15). To test for nutritional programming effects independently of initial differences at the start of HFD feeding, data corresponding to the starch intervention period (*n* = 23-24 per group and sex) and the HFD period (*n* = 12 per group and sex) were analysed separately by repeated measurements two-way ANOVA with Bonferroni's *post hoc* test. In both sexes and periods, both HDD and LDD gained BW, FM, and LM over time ($p<0.0001$). No difference was seen between the two dietary groups, except for the increase in FM in males during the intervention period (F=4.8, $p=0.0344$). However, males fed LDD *vs* HDD during the intervention period gained BW (F=2.9, $p=0.0355$) and FM (F=8.5, $p<0.0001$) at a lower rate, reaching a significant difference in FM at PW 6 ($p<0.0001$). Data shown as mean ± s.d. The dotted line indicates the start of HFD feeding.

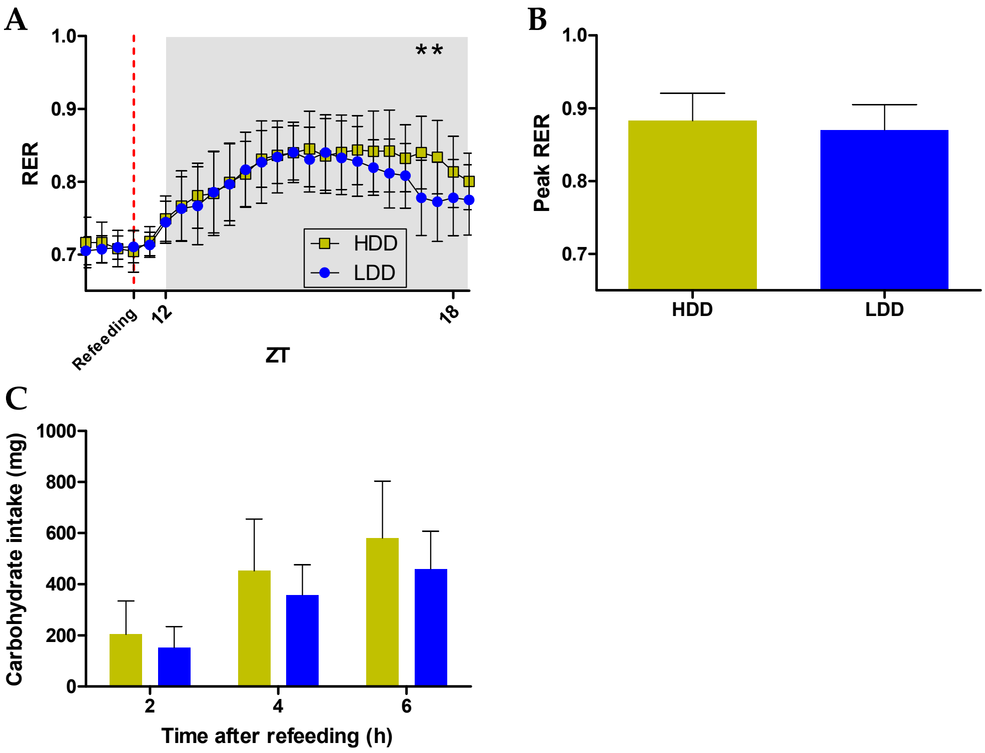

Figure S2. Metabolic flexibility of male mice after 8 weeks of HFD feeding (PW 14). (**A**) RER evolution 1 h before refeeding until 7 h upon *ad libitum* refeeding with a high carbohydrate diet (HDD). Statistical comparison was performed on all data points from the moment of food restriction (additional data points not shown to enhance visualisation). There was a significant interaction of time x post-weaning diet (p=0.0163). (**B**) Mean peak RER values achieved within 7 h after refeeding. (**C**) Cumulative carbohydrate intake calculated from automatic records of food intake after access to refeeding diet. n = 12 per group. Data shown as mean ± s.d. Statistical differences denoted as * p≤0.001.

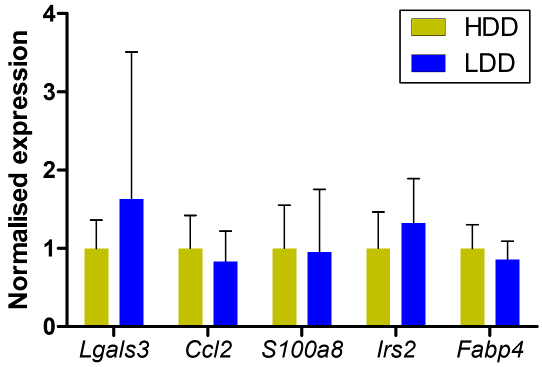

Figure S3. Gene expression in gWAT of females after the HFD feeding period (PW 15). n = 11 for HDD and n = 10 for LDD. Values are given relative to the mean normalised gene expression of the HDD group for each gene and plotted as mean ± s.d. *Lgals3*: lectin, galactose binding, soluble 3; *Ccl2*: chemokine (C-C motif) ligand 2; *S100a8*: S100 calcium binding protein A8 (calgranulin A); *Irs2*: insulin receptor substrate 2; *Fabp4*: fatty acid binding protein 4, adipocyte.

Table S1. Primers used for gWAT gene expression analysis.

Gene symbol	Official Name	Forward primer sequence (5′-3′)	Reverse primer sequence (5′-3′)	Annealing temperature (°C)
Ccl2	chemokine (C-C motif) ligand 2	TTAAAAACCTGGATCGGAACCAA	GCATTAGCTTCAGATTTACGGGT	58.0
Lgals3	lectin, galactose binding, soluble 3	TAATCAGGTGAGCGGCACAG	GCTAAGGCATCGTTAAGCGAAA	58.0
S100a8	S100 calcium binding protein A8 (calgranulin A)	ACTTCGAGGAGTTCCTTGCG	TGCTACTCCTTGTGGCTGTC	60.0
Irs2	insulin receptor substrate 2	GCACCTATGCAAGCATCGAC	GCGCTTCACTCTTTCACGAC	60.0
Fabp4	fatty acid binding protein 4, adipocyte	AATCACCGCAGACGACAGGAAG	TGCCCTTTCATAAACTCTTGTGGAAG	60.0
B2m	beta-2 microglobulin	CCCCACTGAGACTGATACATACGC	AGAAACTGGATTTGTAATTAAGCAGGTTC	60.0
Rps15	ribosomal protein S15	CGGAGATGGTGGGTAGCATGG	ACGGGTTTGTAGGTGATGGAGAAC	60.0
